# PD-L1-specific helper T-cells exhibit effective antitumor responses: new strategy of cancer immunotherapy targeting PD-L1 in head and neck squamous cell carcinoma

**DOI:** 10.1186/s12967-019-1957-5

**Published:** 2019-06-20

**Authors:** Yui Hirata-Nozaki, Takayuki Ohkuri, Kenzo Ohara, Takumi Kumai, Marino Nagata, Shohei Harabuchi, Akemi Kosaka, Toshihiro Nagato, Kei Ishibashi, Kensuke Oikawa, Naoko Aoki, Mizuho Ohara, Yasuaki Harabuchi, Yuji Uno, Hidehiro Takei, Esteban Celis, Hiroya Kobayashi

**Affiliations:** 1 Department of Pathology, Asahikawa Medical University, Midorigaoka-Higashi 2-1-1, Asahikawa, 078-8510 Japan; 2 Department of Otolaryngology, Head and Neck Surgery, Asahikawa Medical University, Midorigaoka-Higashi 2-1-1, Asahikawa, 078-8510 Japan; 3 Respiratory and Breast Center, Asahikawa Medical University Hospital, Midorigaoka-Higashi 2-1-1, Asahikawa, 078-8510 Japan; 4 Department of Pathology, Asahikawa Medical University Hospital, Midorigaoka-Higashi 2-1-1, Asahikawa, 078-8510 Japan; 5 Cancer Immunology, Inflammation and Tolerance Program, Augusta University Georgia Cancer Center, 1120 15th Street, Augusta, GA 30912 USA

**Keywords:** PD-L1, Helper T-cells, Head and neck squamous cell carcinoma, Cancer immunotherapy, Tumor-associated antigen

## Abstract

**Background:**

Head and neck squamous cell carcinoma (HNSCC) originates from squamous epithelium of the upper aerodigestive tract and is the most common malignancy in the head and neck region. Among HNSCCs, oropharynx squamous cell carcinoma (OSCC) has a unique profile and is associated with human papillomavirus infection. Recently, anti-programmed cell death-1 monoclonal antibody has yielded good clinical responses in recurrent and/or metastatic HNSCC patients. Therefore, programmed death-ligand 1 (PD-L1) may be a favorable target molecule for cancer immunotherapy. Although PD-L1-expressing malignant cells could be targeted by PD-L1-specific CD8^+^ cytotoxic T lymphocytes, it remains unclear whether CD4^+^ helper T lymphocytes (HTLs) recognize and kill tumor cells in a PD-L1-specific manner.

**Methods:**

The expression levels of PD-L1 and HLA-DR were evaluated using immunohistochemical analyses. MHC class II-binding peptides for PD-L1 were designed based on computer algorithm analyses and added into in vitro culture of HTLs with antigen-presenting cells to evaluate their stimulatory activity.

**Results:**

We found that seven of 24 cases of OSCC showed positive for both PD-L1 and HLA-DR and that PD-L1_241-265_ peptide efficiently activates HTLs, which showed not only cytokine production but also cytotoxicity against tumor cells in a PD-L1-dependent manner. Also, an adoptive transfer of the PD-L1-specific HTLs significantly inhibited growth of PD-L1-expressing human tumor cell lines in an immunodeficient mouse model. Importantly, T cell responses specific for the PD-L1_241-265_ peptide were detected in the HNSCC patients.

**Conclusions:**

The cancer immunotherapy targeting PD-L1 as a helper T-cell antigen would be a rational strategy for HNSCC patients.

## Background

Head and neck squamous cell carcinoma (HNSCC) originates from squamous epithelium of the upper aerodigestive tract, which includes the nasal and oral cavity, pharynx, and larynx, and is the most common malignancy in the head and neck region with over 600,000 new cases diagnosed each year [1, 2]. Although smoking and alcohol consumption are major risk factors for the development of most HNSCCs, oropharynx squamous cell carcinoma (OSCC) has a unique profile and is associated with human papillomavirus (HPV) infection [3, 4]. Interestingly, patients with HPV-positive oropharyngeal cancer had better 3-year overall survival (OS) and progression-free survival (PFS) rates than those with HPV-negative cancer after treatment with fractionated radiotherapy [5].

Cancer immunotherapy with immune checkpoint inhibitors has been the focus of many studies since the efficacy of immunotherapy targeting the immune checkpoint molecule programmed cell death-1 (PD-1) and its ligand PD-L1 was demonstrated [6–11]. PD-L1 plays an important role in immune regulation by binding to PD-1 expressed on effector T-cells to induce apoptosis or anergy in order to prevent autoimmune disease [12, 13]. Furthermore, tumor cells also take advantage of PD-L1 to escape from antitumor immune responses. Indeed, high PD-L1 expression is frequently found in tumor tissues and correlates with poor prognosis [14–17].

Therefore, blockade of the PD-1/PD-L1 signaling pathway by using specific antibodies to PD-1, such as nivolumab, yielded remarkable clinical responses in metastatic melanoma [9], non-small cell lung cell cancer [18], and renal cell carcinoma [19]. The efficacy of immunotherapy, particularly blockade of the PD-1/PD-L1 pathway, in HNSCC patients was recently demonstrated [20], although HNSCC was initially recognized as an immunosuppressive tumor from the perspective of lower lymphocyte count, spontaneous apoptosis of cytotoxic T lymphocytes (CTLs), and poor antigen-presenting function in patient blood samples [21]. Furthermore, 6-month OS and PFS rates of recurrent and/or metastatic HNSCC patients treated with pembrolizumab, an anti-PD-1 monoclonal antibody, were 23% and 59%, respectively, showing a favorable response similar to single-drug cetuximab [22, 23].

Based on this evidence, PD-1/PD-L1 signaling plays a critical role in suppressing immune responses against HNSCC as well, suggesting that immunotherapy targeting PD-L1-expressing HNSCC cells by acquired immunity would be a rational antitumor strategy. Indeed, PD-L1 is a favorable target molecule for cancer immunotherapy and PD-L1-expressing malignant cells were killed by PD-L1-specific CD8^+^ CTLs in a PD-L1-dependent manner [24, 25]. However, there are no reports about PD-L1-specific CD4^+^ helper T lymphocytes (HTLs).

In cancer immunotherapy, HTLs not only support CTLs by promoting effector functions and long-term survival but also have direct cytotoxicity against cancer cells via effector cytokines [26]. Thus, we hypothesized that PD-L1-specific HTLs are also required for enhancing effective antitumor immunotherapy.

In the current study, we defined the helper epitope peptide in PD-L1 for inducing PD-L1-specific HTLs from peripheral blood of healthy donors for the first time. PD-L1-specific HTLs produced effector cytokines and demonstrated cytotoxicity against PD-L1-expressing tumor cells. Remarkably, PD-L1-specific HTLs adoptively transferred into immunodeficient mice significantly inhibited growth of PD-L1-positive human lung carcinoma. Also, specific T-cells to the peptide were observed in the HNSCC patients. These findings suggest that PD-L1 could be a promising antitumor target and immunotherapy using PD-L1-specific HTLs would be a rational approach for patients with HNSCC.

## Methods

### Cell lines and mice

HNSCC cell lines Sa-3 [gingival squamous cell carcinoma (SCC), HLA-DR9/10], HSC-3 (tongue SCC, HLA-DR15/15), HSC-4 (tongue SCC, HLA-DR1/4), and human lung large cell carcinoma cell line Lu65 (HLA-DR4/15) were supplied by RIKEN BioResource Center (Tsukuba, Ibaraki, Japan). HNSCC cell line HPC-92Y (hypopharyngeal SCC, HLA-DR4/9) was kindly provided by Dr. S. Yanoma (Yokohama Tsurugamine Hospital, Yokohama, Japan). Tumor cell line SAS (tongue SCC, HLA-DR9/15) was purchased from ATCC (Manassas, VA). L-cells (mouse fibroblasts) expressing transfected HLA class II molecules were obtained from Dr. R. Karr (Karr Pharma, St. Louis, MO) and Dr. T. Sasazuki (Kyushu University, Fukuoka, Japan). All cell lines were maintained in tissue culture as recommended by the supplier. BALB/c-nu mice (female, 8 to 10-week-old) were purchased from Charles River Laboratories Japan, Inc. (Yokohama, Japan). All cell lines were meticulously cultured and used up within 6 months although no authentication assay was performed for all cell lines used. Mice were maintained and handled according to the protocols approved by the Asahikawa Medical University Institutional Animal Care and Use Committee.

### Flow cytometry

HLA-DR and PD-L1 expression on tumor cell surfaces were evaluated by flow cytometry using anti-HLA-DR monoclonal antibody (mAb) (G46-6) conjugated with fluorescein isothiocyanate (BD Pharmingen) and anti-PD-L1 mAb (MIH) conjugated with phycoerythrin (eBioscience, Thermo Fisher Scientific). Mouse IgG2a antibody (MOPC-173) and mouse IgG1 antibody (MOPC-21) were purchased from BioLegend and used as isotype controls for HLA-DR and PD-L1, respectively. All tumor cell lines were treated with or without 500 IU/ml interferon gamma (IFN-γ) for 48 h before analysis. The samples were analyzed using the BD Accuri C6 flow cytometer and software (BD Biosciences).

### Western blotting

Tumor cell lines (1 × 10^6^) were washed in phosphate-buffered saline (PBS) and lysed in LDS sample buffer (Invitrogen, Thermo Fisher Scientific). The cell lysate was subjected to electrophoresis in a 4–12% NuPAGE Bis–Tris SDS-PAGE gel (Invitrogen, Thermo Fisher Scientific) under reducing condition and transferred to an Immobilon-P membrane (Merck Millipore). The membrane was blocked in PBS containing 0.01% Tween 20 and 5% non-fat dry milk for 1 h at room temperature and incubated with polyclonal rabbit anti-human PD-L1 (E1L3N, Cell Signaling Technology) diluted 1:1000 in blocking buffer for overnight at 4 °C, or anti-β-actin mAb (C4, Santa Cruz Biotechnology) diluted 1:2000 in blocking buffer as the control for 1 h at room temperature. After washing, the membrane was incubated with horseradish peroxidase-labeled sheep anti-rabbit or anti-mouse IgG and visualized using the Amersham ECL Prime Western Blotting Detection System (GE Healthcare Life Sciences).

### Synthetic peptides

We used the two computer-based algorithms the Immune Epitope Database Analysis Resource (IEDB, https://www.iedb.org/) [27] and SYFPEITHI (http://www.syfpeithi.de/) [28] for identifying potential HLA-DR (DRB1*0101, DRB1*0401, DRB1*0701, DRB1*1101, and DRB1*1501)-binding amino acid sequences of PD-L1. The cut-off values for selecting a potential epitope peptide were set over 22 and under 4 in IEDB and SYFPEITHI, respectively. The peptides that showed high scores in both algorithms were selected as possible epitopes. As a result, we selected PD-L1-derived peptides PD-L1_189-214_ (KLFNVTSTLRINTTTNEIFYCTFRRL) and PD-L1_241-265_ (LVILGAILLCLGVALTFIFRLRKGR) and commercially synthesized them (GenScript). PADRE peptide (aK-Cha-VAAWTLKAAa, a = D-alanine; and Cha = I-cyclohexylalanine) was used as a positive control for activating HTLs.

### Knockdown of PD-L1 using siRNA

Tumor cell lines Sa-3, HSC-4, SAS, and Lu65 were transfected with PD-L1 siRNA using Lipofectamine RNAi MAX Reagent (Invitrogen, Thermo Fisher Scientific). We used a mixture of three types of siRNA, which were (5′ to 3′) GAG GAA GAC CUG AAG GUU CAG CAU A, CCU ACU GGC AUU UGC UGA ACG CAU U, and UGA UAC ACA UUU GGA GGA GAC GUA A. Stealth siRNA duplex for targeting PD-L1 and recommended Stealth siRNA negative control duplex for medium GC content (catalog number: 12935112) were purchased from Invitrogen. Transfected tumor cells were used for assay after 96-h incubation of transfection.

### In vitro generation of PD-L1-specific CD4^+^ HTLs

The procedure for the induction of tumor-specific CD4^**+**^ HTLs has been described in detail previously [29]. Briefly, monocytes and CD4^+^ T-cells were purified from peripheral blood mononuclear cells (PBMCs) using MACS microbeads for CD14 and CD4, respectively (Miltenyi Biotech). To prevent the antibodies from binding non-specifically, we used the FcR blocking reagent (Miltenyi Biotech). Monocytes were differentiated into dendritic cells (DCs) using granulocyte macrophage colony-stimulating factor (GM-CSF) (50 ng/ml) and interleukin (IL)-4 (1000 IU/ml). PD-L1 peptide-pulsed DCs (3 μg/ml for 3 h at room temperature) were co-cultured with autologous CD4^+^ T-cells in 96-well flat-bottomed culture plates. Seven days later, the CD4^+^ T-cells were restimulated in individual microcultures with PD-L1 peptide-pulsed γ-irradiated autologous PBMCs (3 μg/ml), and 2 days later, recombinant human IL-2 (10 IU/ml) was added. After the two cycles of peptide stimulation, PD-L1-specific T-cell lines were expanded by weekly restimulation with cognate peptides (3 μg/ml)-pulsed irradiated autologous PBMCs. Production levels of IFN-γ (BD Pharmingen) and Granzyme B (MABTECH) in culture supernatants were determined by ELISA kits according to the manufactures’ instructions. We measured the absorption at 450 nm by GloMax Discover Microplate Reader (Promega). Three percent of human male AB serum (Innovative Research)-supplemented AIM-V medium (Invitrogen, Carlsbad, CA) was used as complete culture medium for all experiments. All blood materials were acquired after informed consent was appropriately obtained.

### Addressing PD-L1-specific responses with established CD4^+^ T-cell lines

CD4^+^ HTLs (1–1.5 × 10^5^) were co-cultured with irradiated antigen-presenting cells (APCs) in the presence of various concentrations of PD-L1 peptides in 96-well culture plates. Autologous PBMCs (1.5 × 10^5^), HLA-DR-expressing L-cells (3 × 10^4^), tumor cell lines (3 × 10^4^), or DCs (5 × 10^3^) were used as APCs. HNSCC cell lines were treated with 500 U/ml IFN-γ for 48 h to upregulate HLA-DR expression, and then IFN-γ was removed before assay. To determine antigen specificity and HLA-DR restriction, anti-HLA-DR mAb L243 (IgG2a, prepared from the supernatants of hybridoma HB-55 obtained from ATCC) and anti-HLA-A/B/C mAb W6/32 (IgG2a; ATCC) were added to the culture at 10 μg/ml for a 48-h incubation period. Supernatants were collected for evaluation of IFN-γ and Granzyme B production using the ELISA kits as mentioned above.

### Measurement of peptide-specific responses in HNSCC patients

PBMCs (2–3 × 10^5^) of patients with HNSCC were cultured with PD-L1_241-265_ and PADRE peptides (10 μg/mL) in 96-well plates as described previously [30]. Ten days after peptide stimulation, IFN-γ in the supernatants was assessed by ELISA.

### Cytotoxicity assay

Cytotoxic activity of CD4^+^ HTL lines was measured by flow cytometry using the BD Accuri C6 flow cytometer and software (BD Biosciences). HSC-4, Lu65 (matched HLA-DR with PD-L1-specific HTLs), and SAS (unmatched HLA-DR with PD-L1-specific HTLs) were labeled by using the CellTrace™ CFSE Cell Proliferation Kit (Life Technologies) after pretreatment with or without IFN-γ (500 U/ml) for 24 h. After co-culture with PD-L1-specific HTLs for 6 h, tumor cells were collected, and dead cells were detected with 7-AAD viability staining solution (BioLegend). Cytotoxicity of PD-L1-specific HTLs was assessed in various effector/target cell (E:T) ratios (0:1, 10:1, and 30:1).

### Assessing antitumor effects of PD-L1-specific HTLs in immunodeficient mice

Lu65 (HLA-DR4^+^) and Sa-3 (HLA-DR4^−^) cell lines were intradermally injected into BALB/c-nu mice on day 0. Human IFN-β (BioLegend) (5000 U/shot) was intratumorally injected into the mice on days 19 and 26. PD-L1_241-265_-specific HTLs or control CD4^+^ T-cells (1–2 × 10^6^) were intravenously injected into the mice on days 20 and 27. The control T-cells were stimulated and expanded in vitro with anti-CD3 mAb (OKT3; Lymactin-T, Cell Science & Technology Inst., Inc.). Tumor size was measured twice a week.

### H&E staining and immunohistochemistry

Formalin-fixed tissue sections were subjected to H&E staining according to a standard protocol. Immunohistochemistry was performed using the Envision™ HRP System (Agilent Technologies Dako) as described previously [31]. Formalin-fixed, paraffin-embedded tumor tissue sections were acquired from oropharynx cancer patients. Ventana PD-L1 (SP263) rabbit mAb (1:1 dilution) (Roche) or Dako HLA-DR Antigen, Alpha-Chain (TAL.1B5) mouse mAb (1:80 dilution) (Agilent) were used as primary antibody against PD-L1 and HLA-DR, respectively. The institutional ethics committee approved this study, and written informed consent was obtained from all patients who provided tissue samples. Distribution of HLA-DR staining was graded by percentage of tumor cells that were positive and then divided into quartiles as: 0–9%; negative, 10–25%; weak, 26–45%; moderate, and 51–100%; strong as previously reported [32].

### Statistical analysis

Values shown are the means of triplicate determinations (Figs. 2, 3, 4, 5) or six mice (Fig. 6). All data were analyzed by Student’s t-test, one-way ANOVA with the Holm post hoc test, or unpaired t test. P values < 0.05 were considered statistically significant.

## Results

### Various expression levels of PD-L1 and HLA-DR in oropharynx squamous cell carcinoma

**Fig. 1:**
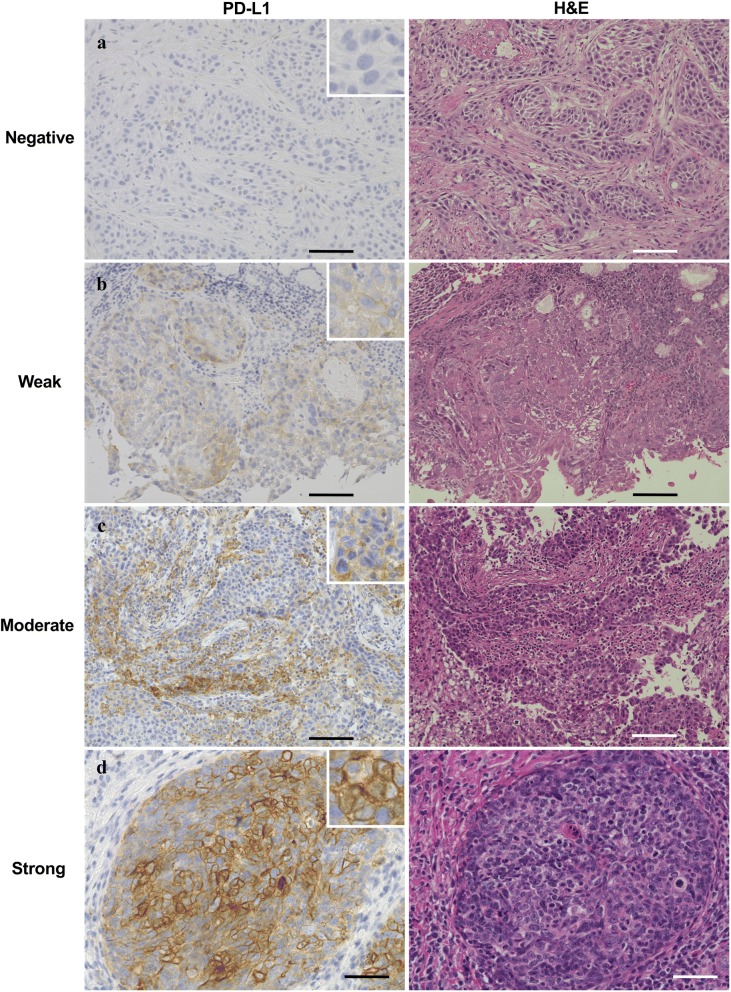

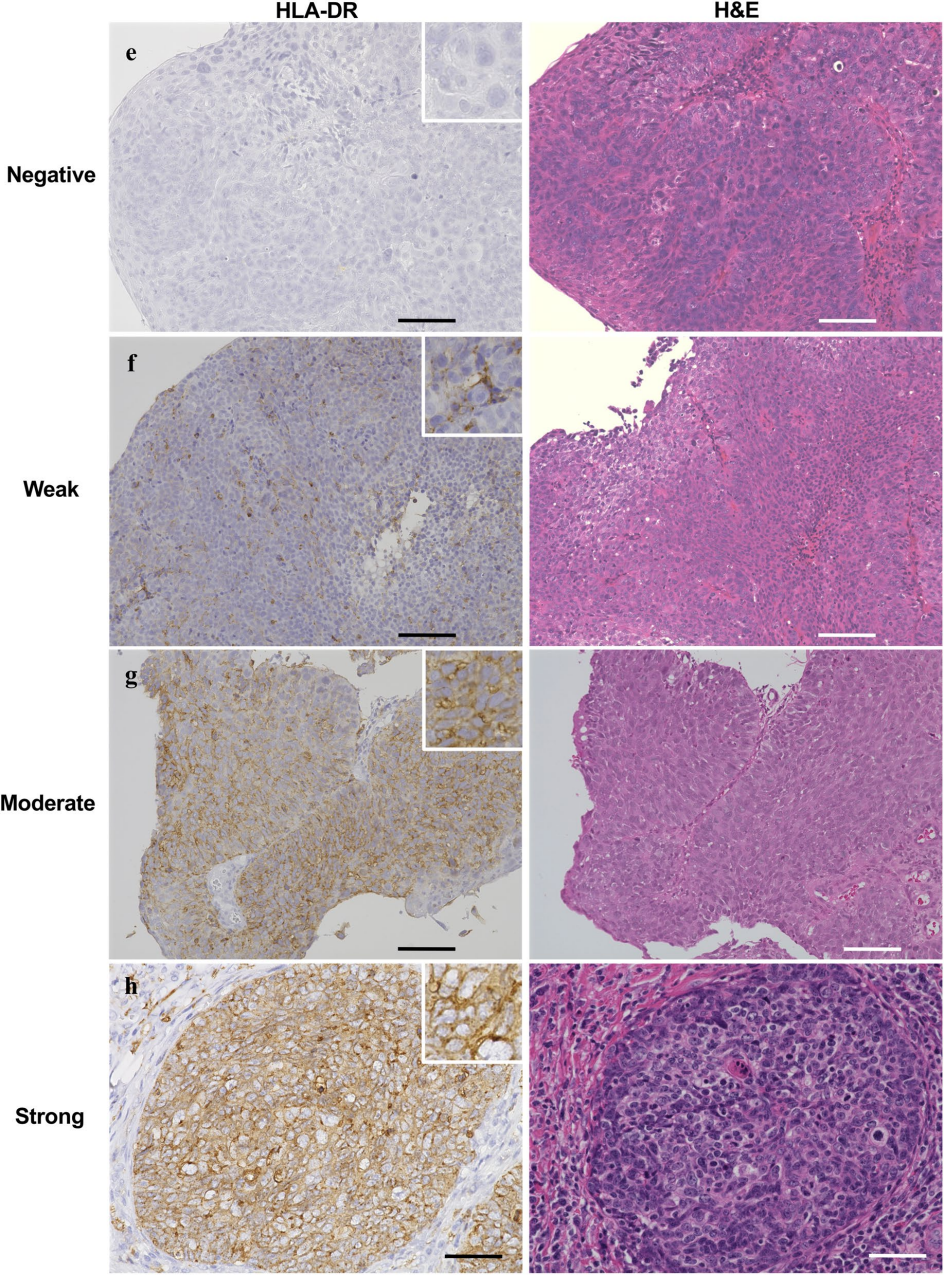
Expression levels of PD-L1 and HLA-DR in oropharynx squamous cell carcinoma tissue. Tissue specimens of patient with oropharynx squamous cell carcinoma were classified into four groups by immunostaining intensity for PD-L1 or HLA-DR: **a**, **e** negative staining; **b**, **f** weak staining; **c**, **g** moderate staining; **d**, **h** strong staining. H&E staining was shown in the right. Representative images are shown. Scale bar is 100 μm

Immunohistochemical analyses were performed to assess PD-L1 and HLA-DR expression in oropharynx cancer tissues from 24 patients treated at Otolaryngology, Head and Neck Surgery, Asahikawa Medical University. Each specimen was blindly checked by three pathologists and classified into four types according to immunostaining intensity for PD-L1 or HLA-DR as follows: negative, weak, moderate, or strong staining. PD-L1 expression in specimens with 5% tumor cell membranous staining and HLA-DR expression in specimens with 10% tumor cell membranous staining were considered “positive”, as previously reported [33–35]. As shown in Fig. 1a–d, immunostaining for PD-L1 showed that 8 (33.3%), 4 (16.7%), 4 (16.7%), and 8 (33.3%) cases of all 24 cases showed negative, weak, moderate, and strong staining, respectively. Moreover, among PD-L1-positive cases, we found that 13 (81.3%) cases showed focal staining between tumor cell nests and stroma, whereas 3 (18.6%) cases showed diffuse staining in tumor cell nests for PD-L1 (data not shown).

For HLA-DR immunostaining, 16 (66.7%), 2 (8.3%), 3 (12.5%), and 3 (12.5%) cases of 24 cases were negatively, weakly, moderately, and strongly stained with anti-HLA-DR antibody, respectively (Fig. 1e–h). Seven of 24 cases (29.2%) were positive for both PD-L1 and HLA-DR. Data are summarized in Table 1. There were not any differences between the expression patterns and staging of the patients (data not shown). H&E staining was also performed as shown in Fig. 1.

**Table 1:** Expression status of PD-L1 and HLA-DR in patients with oropharynx squamous cell carcinoma

	PD-L1				Total for HLA-DR
	Negative	Weak	Moderate	Strong	
HLA-DR					
Negative	7	2	2	5	16
Weak	0	0	1	1	2
Moderate	1	2	0	0	3
Strong	0	0	1	2	3
Total for PD-L1	8	4	4	8	24

### Identification of helper peptides for PD-L1 and generation of PD-L1-specific helper T-cell lines

**Fig. 2:**
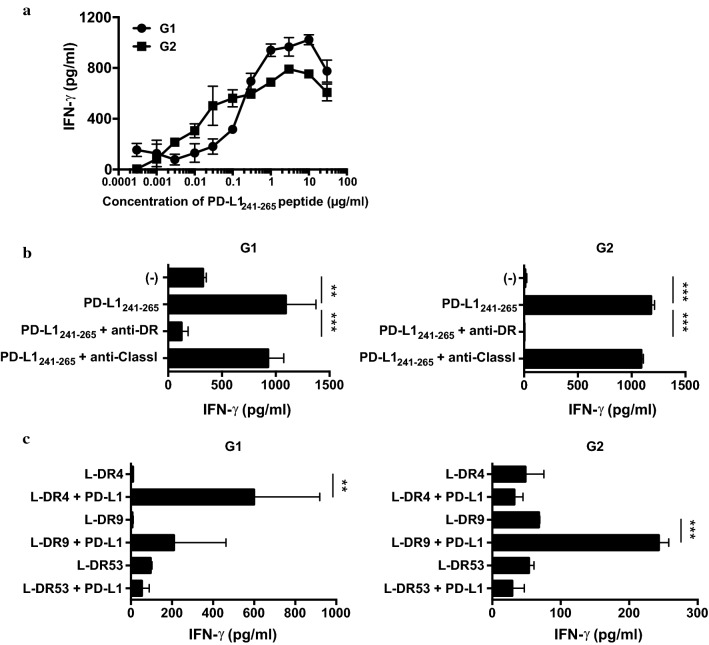
Generation of PD-L1_241-265_ peptide-specific CD4^+^ T-cells. **a** Two CD4^+^ T-cell lines (G1 and G2) specific for PD-L1_241-265_ peptide were evaluated for their IFN-γ production in response to irradiated autologous PBMCs in the presence of various concentration of PD-L1_241-265_ peptide. **b** HLA restriction of each PD-L1_241-265_ -reactive CD4^+^ T-cell line was evaluated by using anti-HLA-DR mAb L243 and anti-HLA class I mAb W6/32 (negative control). **c** Each CD4^+^ T-cell line was cocultured with PD-L1_241-265_ peptide-pulsed L-cells expressing individual HLA-DR allele. Supernatants were collected after 48 h and analyzed for IFN-γ release by ELISA. Bars and error bars indicate the mean and SD of triplicate determinations, respectively. Each data is representative of two separate experiments

To identify a helper epitope peptide for PD-L1, PD-L1_189-214_ and PD-L1_241-265_ were selected as candidates for potential MHC class II binding peptide sequences by using computer-based algorithms. CD4^+^ T-cells were purified from PBMCs of healthy donors and stimulated with each PD-L1 peptide onto autologous DCs and restimulated with PD-L1 peptide-pulsed γ-irradiated autologous PBMCs repeatedly once a week. We obtained PD-L1_241-265_-specific CD4^+^ T-cell lines from two healthy donors (G1: HLA-DR4/DR53 and G2: HLA-DR9/DR53). No reactions of CD4^+^ T-cells to PD-L1_189-214_ peptide were detected. Each PD-L1_241-265_-specific CD4^+^ T-cell line released IFN-γ in a dose-dependent manner (Fig. 2a). To define their HLA-DR restriction, we evaluated the reactivity of PD-L1_241-265_-specific CD4^+^ T-cells to autologous PBMCs in the presence of PD-L1_241-265_ peptide by using anti-HLA-DR or anti-HLA class I mAbs. The IFN-γ production of both PD-L1_241-265_-specific CD4^+^ T-cell lines were inhibited by anti-HLA-DR mAbs, but not by anti-HLA class I mAbs, suggesting that peptide recognition of both PD-L1_241-265_-specific CD4^+^ T-cell lines was restricted to HLA-DR (Fig. 2b). Furthermore, we assessed the reactivity of PD-L1_241-265_-specific CD4^+^ T-cell lines using mouse fibroblasts (L-cells) transfected with HLA-DR allele gene as APCs. The T-cell line G1 responded to L-DR4 cells and the T-cell line G2 responded to L-DR9 cells, indicating that these T-cell lines G1 and G2 were restricted to HLA-DR4 and HLA-DR9, respectively (Fig. 2c).

### Direct tumor recognition by PD-L1-specific HTLs

**Fig. 3:**
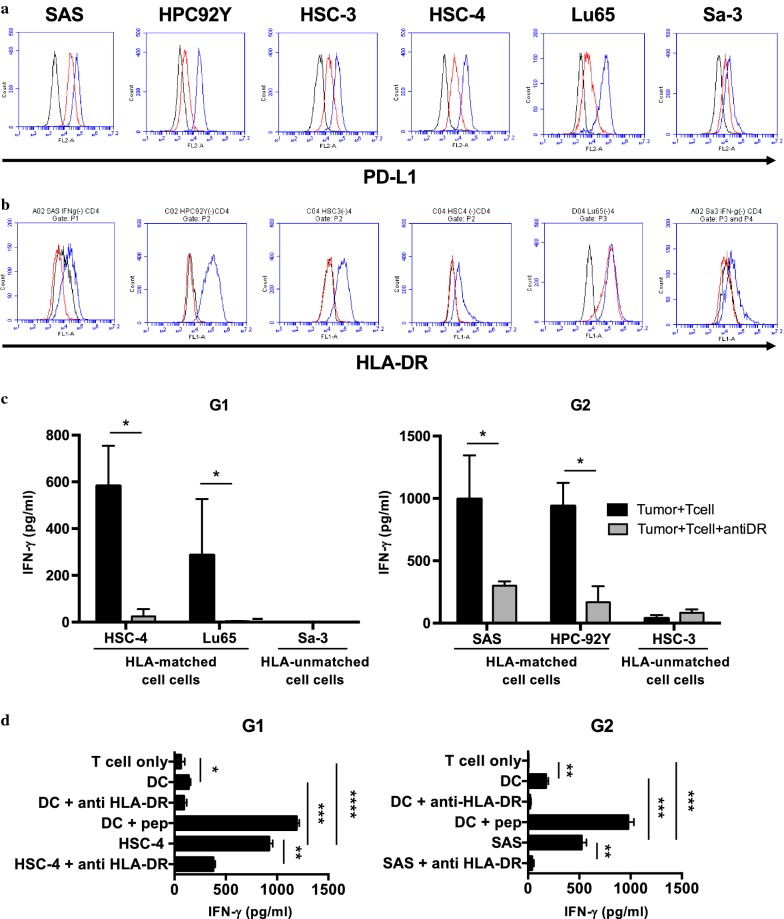
Recognition of PD-L1_241-265_-specific CD4^+^ T-cell lines against tumor cell lines and DCs expressing PD-L1. Expressions of PD-L1 and HLA-DR on tumor cell lines were examined by flow cytometry after treatment with IFN-γ for 48 h. Representative flow histograms for **a** PD-L1 and **b** HLA-DR expressions were shown in upper panels and lower panels, respectively. Black: isotype control (MOPC-21 for PD-L1 and MOPC-173 for HLA-DR), Red: untreatment, Blue: IFN-γ treatment (500 U/ml). **c** PD-L1_241-265_-specific CD4^+^ T-cell lines were cocultured with HLA-DR-matched and unmatched tumor cell lines expressing PD-L1 with/without anti-HLA-DR mAbs as indicated. Supernatants were collected after 48 h and analyzed for IFN-γ by ELISA. **d** The reactivity of PD-L1_241-265_-specific CD4^+^ T-cells (G1 and G2) to autologous DCs and tumor cell lines. DCs and tumor cells (HSC4 or SAS) were treated with IFN-γ (500 U/ml, 48 h), and were cocultured with each PD-L1_241-265_-specific CD4^+^ T-cells for 24–48 h. Anti-HLA-DR antibody was used for blocking HLA-DR-specific reaction. Supernatants were collected and analyzed by ELISA for IFN-γ release. Bars and error bars indicate the mean and SD of triplicate determinations, respectively. Each data is representative of two separate experiments

To address whether PD-L1_241-265_-reactive CD4^+^ T-cell lines would directly target PD-L1-expressing tumor cells, PD-L1 and HLA-DR expressions on tumor cell lines were evaluated. Although PD-L1 was primarily expressed, HLA-DR required IFN-γ treatment for their expressions in HNSCC cell lines (SAS, HPC-92Y, HSC-3, and HSC-4), but not lung large cell carcinoma cell line: Lu65 (Fig. 3a, b). Thus, we co-cultured PD-L1_241-265_-specific CD4^+^ T-cells with the HLA-DR-matched tumor cell lines expressing PD-L1. Expectedly DR4-restricted G1 cells released IFN-γ against HSC-4 (HLA-DR1/DR4) and Lu65 (HLA-DR4/DR15) cells in an HLA-DR-dependent manner. Also, DR9-restricted G2 cells responded to tumor cell lines positive for HLA-DR9 (SAS and HPC-92Y). Both CD4^+^ T-cell lines did not respond to HLA-unmatched tumor cell lines (Fig. 3c), suggesting that our defined PD-L1_241-265_ peptide could efficiently stimulate PD-L1-expressing tumor-reactive HTLs. Because PD-L1 is also expressed on non-tumoral cells such as placental cells and DC, we assessed whether PD-L1_241-265_-specific CD4^+^ T-cells (G1 and G2) respond to DCs expressing PD-L1. Although the response of the T-cell lines to peptide-loaded autologous DCs appeared higher than tumor cell lines, unloaded DCs didn’t stimulate PD-L1_241-265_-specific CD4^+^ T-cells as much as tumor cell lines (Fig. 3d).

**Fig. 4:**
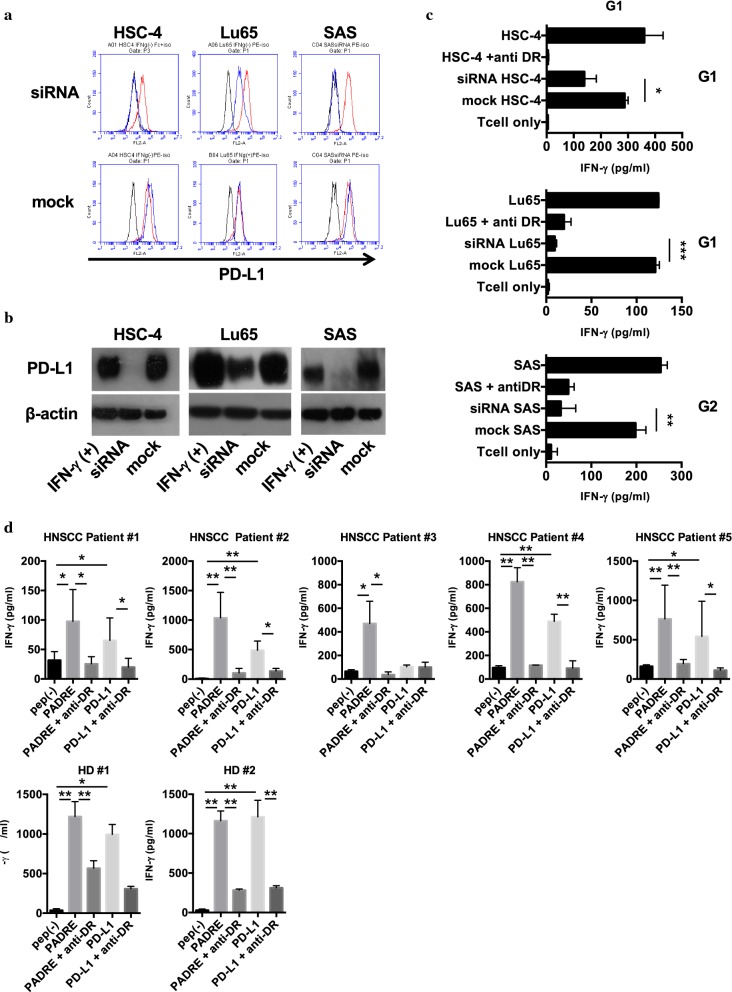
The diminished responses of PD-L1_241-265_-specific CD4^+^ T-cell lines against tumor cell lines transfected with PD-L1-specific siRNA. **a** Expression levels of PD-L1 on tumor cell lines were examined by flow cytometry. Black: isotype control, Red: PD-L1 on parental cell lines, Blue: PD-L1 on tumor cell lines transfected with PD-L1-specific siRNA (upper panels) or mock siRNA (lower panels). Each tumor cells was treated with IFN-γ (500 U/ml) for 48 h. **b** Evaluation of PD-L1 in tumor cell lines was performed by Western blot. **c** PD-L1 _241-265_-specific CD4^+^ T-cell lines were co-cultured with HLA-DR-matched tumor cell lines after transfection of PD-L1-targeting siRNA or mock. Supernatants were collected and analyzed by ELISA for IFN-γ release. The results shown are representative of two separate experiments. **d** The reactivity of PBMCs of five HNSCC patients and two healthy donors (HD) against PD-L1_241-265_ peptides was assessed. Isolated PBMCs were cultured in the presence of PD-L1_241-265_ or PADRE peptides (10 μg/mL) for 10 days. Supernatants were collected and analyzed by ELISA for IFN-γ release. Bars and error bars indicate the mean and SD of triplicate determinations, respectively. Each data is representative of two separate experiments

We next assessed whether the recognition of PD-L1_241-265_-specific CD4^+^ T-cells is surely dependent on PD-L1 expression in tumor cells. Tumor cell lines were transfected with PD-L1-specific siRNA or mock siRNA (negative control) and then treated with or without IFN-γ (500 U/ml) for 48 h. Downregulated expression of PD-L1 was confirmed by flow cytometry and western blotting (Fig. 4a, b). Cytokine productions in both PD-L1_241-265_ -specific CD4^+^ T-cell lines were significantly diminished against PD-L1-specific siRNA-transfected tumor cell lines compared with mock-transfected control tumor cell lines (Fig. 4c), suggesting that PD-L1_241-265_-specific CD4^+^ T-cell lines certainly recognize tumor cell lines expressing PD-L1.

### Recognition of PD-L1 peptides by PBMCs from HNSCC patients

It is important to confirm whether the PD-L1_241-265_ peptide also shows antigenic activity in patients with HNSCC for clinical applications because HNSCC has been reported to dysregulate immune cells [21]. Thus, we performed a short-term culture of PBMC derived from 5 patients with HNSCC and 2 healthy donors in the presence of the PD-L1_241-265_ peptide because the volumes of blood obtained from these patients were small. As shown in Fig. 4d, substantial T-cell responses to PD-L1_241-265_ peptides were observed not only in healthy donors but also in HNSCC patients (4/5 tested). This means that the precursor of PD-L1_241-265_-specific CD4^+^ T-cells surely exists in HNSCC patients.

### Cytotoxic activity and antitumor effect of PD-L1-specific helper T-cell lines

**Fig. 5:**
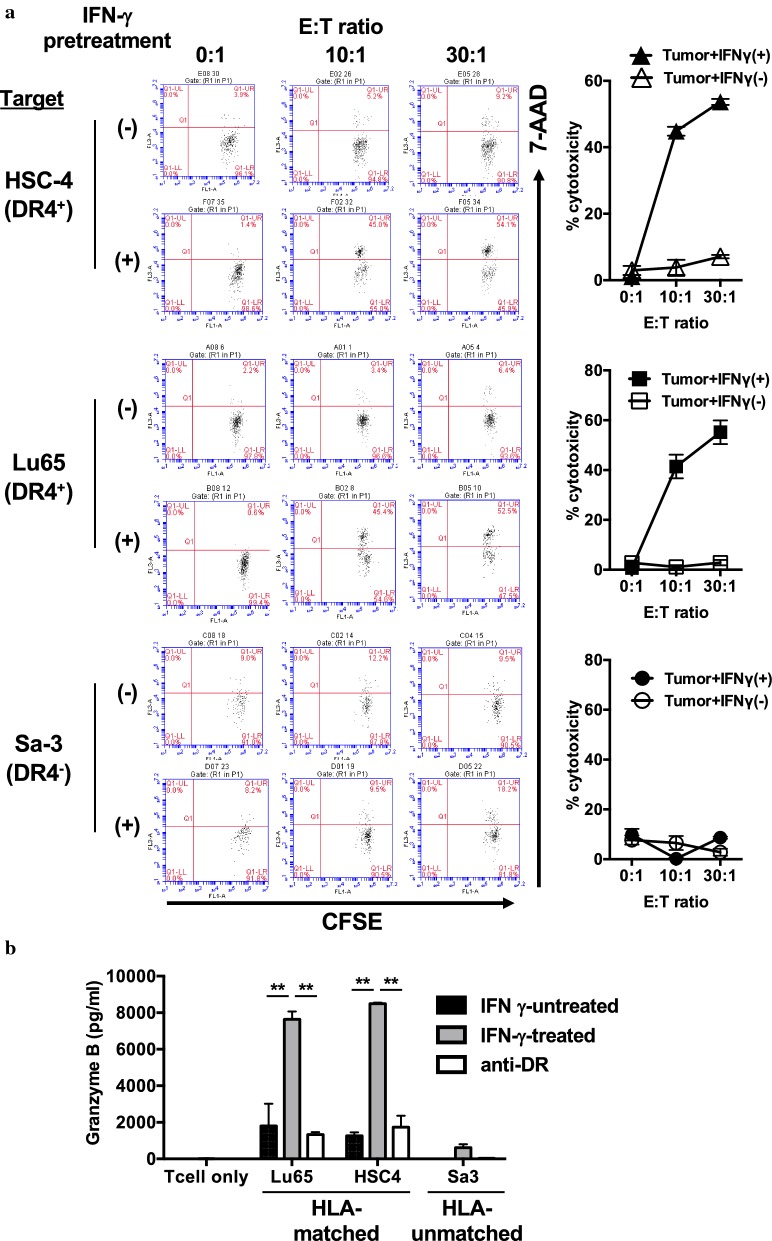
The cytotoxicity of PD-L1_241-265_-specific CD4^+^ T-cell lines against HLA-matched tumor cell lines expressing PD-L1. **a** PD-L1_241-265_-specific CD4^+^ T-cell lines (G1; HLA-DR4-restricted) were cocultured with CFSE-labeled tumor cell lines HSC-4 and Lu65 expressing PD-L1 pre-treated with or without IFN-γ (500 U/ml). HLA-DR-unmatched cell line Sa-3 was used as a negative control. After 6 h of coculture, the cells were collected to evaluate percentages of dead cells by using 7-AAD with flow cytometry. E:T (Effector: Target cells) ratio was 0:1, 10:1, and 30:1. Left panels show representative data of flow cytometry analysis. Right panels show the averages of cytotoxicity of the G1 cell lines against each tumor cell lines. Each result is representative of two separate experiments. **b** PD-L1_241-265_-specific CD4^+^ T-cell lines (G1; HLA-DR4-restricted) were cocultured with tumor cell lines HSC-4 and Lu65 pre-treated with or without IFN-γ (500 U/ml). HLA-DR-unmatched cell line Sa-3 was used as a negative control. Supernatants were collected and analyzed by ELISA for Granzyme-B release after 24 h of coculture. Bars and error bars indicate the mean and SD of triplicate determinations, respectively. Each result is representative of two separate experiments

We then assessed the cytotoxicity of PD-L1_241-265_-specific CD4^+^ T-cell line G1 against PD-L1-expressing tumor cell lines. Although the T-cell line G1 showed highly cytotoxic activity against HLA-DR-matched tumor cell lines HSC-4 and Lu65, but not against HLA-DR-unmatched tumor cell line Sa-3, pretreatment of tumor cell lines with IFN-γ was required for upregulating both HLA-DR and PD-L1 (Fig. 5a). Also, PD-L1_241-265_-specific CD4^+^ T-cell line G1 produced granzyme B against HLA-DR-matched tumor cell lines pretreated with IFN-γ (HSC-4 and Lu65), but not against Sa-3 (Fig. 5b).

### Antitumor effects of PD-L1-specific HTLs in immunodeficient mice

**Fig. 6:**
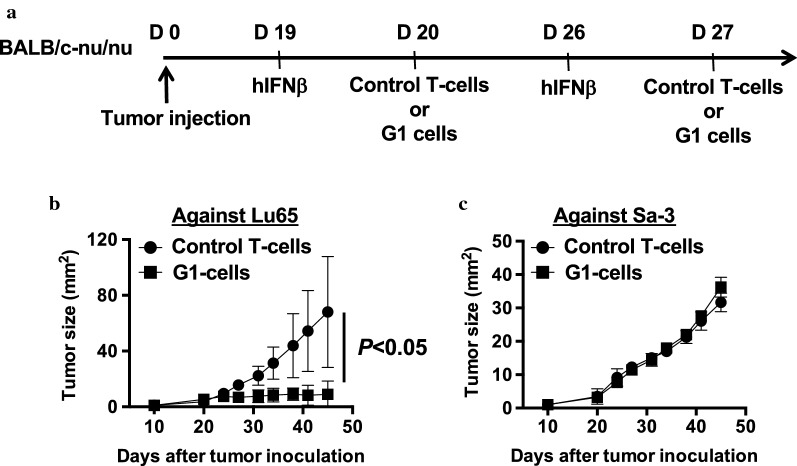
Anti-tumor effect of PD-L1_241-265_-specific CD4^+^ T-cell lines in vivo mouse model. **a** Scheme of adoptive transfer model. Tumor size was monitored in nude mice inoculated with **b** HLA-matched Lu65 (n = 6) and **c** HLA-unmatched Sa-3 (n = 6). Error bars indicate the SD of each mouse in the group. The graphs are representative of two separate experiments. Statistical analysis was performed in Prism using unpaired t test

Having observed that PD-L1-specific G1 HTLs have ability to kill tumor cells, we further evaluated whether PD-L1_241-265_-specific CD4^+^ T-cells exhibit an antitumor effect in vivo setting using an immunodeficient mouse. On day 0, 5 × 10^5^ Lu65 or Sa-3 cell lines were intradermally injected into BALB/c-nu mice. On days 19 and 26, we intratumorally injected human IFN-β (5000 U/shot) into the mice because type I IFNs not only upregulate PD-L1 molecules but also induce T cell-recruiting chemokines in both immune cells and tumor cells [36]. On days 20 and 27, 1–2 × 10^6^ PD-L1_241-265_-specific G1 cells or control T-cells were intravenously injected into the mice (Fig. 6a). In Lu65-bearing mice, PD-L1-specific HTLs G1 cells significantly inhibited tumor growth compared with control T-cells (Fig. 6b). In contrary, PD-L1-specific HTLs did not show any anti-tumor effects in Sa-3-bearing mice (Fig. 6c).

## Discussion

In the current study, we newly identified a PD-L1-derived helper epitope peptide (PD-L1_241-265_) and demonstrated the potential use of PD-L1 as a tumor-associated antigen (TAA). PD-L1_241-265_ efficiently stimulated and expanded PD-L1-specific HTLs from peripheral blood of healthy donors and patients with HNSCC. PD-L1_241-265_-specific HTLs showed not only cytokine production but also cytotoxicity against tumor cells in a PD-L1-specific manner. Surprisingly, adoptively transfer of PD-L1_241-265_-specific HTLs into immunodeficient mice significantly inhibited growth of PD-L1-expressing human lung carcinoma. Because 7 of 24 cases (29.2%) of OSCC were positive for both PD-L1 and HLA-DR expressions, PD-L1-targeted immunotherapy using PD-L1_241-265_ peptide would be applicable to almost 30% of OSCC patients.

PD-L1 is expressed in many malignancies including HNSCCs, in which PD-L1 expression ranges from 46 to 91% [34, 37, 38]. Although it has been reported that there was a strong correlation between PD-L1 expression and OS in HNSCC [39], our immunohistological analyses showed no correlation between them. This discrepancy may be caused by our small sample size (24 cases).

OSCC is distinguished from other HNSCCs in terms of etiological cause, which includes smoking, alcohol consumption, and especially HPV infection. Indeed, in a worldwide systematic review, the percentage of HPV-positive tumors was significantly higher (35.6%) in OSCC than in oral and laryngeal SCCs (23.5% and 24.0%, respectively) [40]. Moreover, OS of patient with OSCC was significantly longer in HPV-positive cases than in HPV-negative cases in contrast to patients with other HNSCCs (oral cavity, hypopharyngeal, and laryngeal cancer) [41]. In our immunohistological analyses, we also found significantly higher PD-L1 expression levels in the p16-positive group compared with the p16-negative group [12/15 (80.0%) vs 4/9 (44.4%), p = 0.014, data not shown]. Similarly, Lyfold-Pike et al. reported that HPV-positive tumors expressed PD-L1 more frequently than HPV-negative tumors [14/20 (70%) vs. 2/7 (29%)] [35]. However, the relation between PD-L1 expression and HPV status remains unclear because some reports have shown no such association [34, 37, 38]. Therefore, this association must be further elucidated. Among HPV-positive OSCC, PD-L1-positive cases also showed significantly longer PFS than PD-L1-negative cases [37]. This finding suggests that while PD-L1 expression on tumor cells is generally considered to indicate poor prognosis, HPV and PD-L1 status might be good markers for prognosis in patients with OSCC.

Although PD-L1 is regarded as a functional molecule that sends a suppressive signal to effector T-cells, it could also be a target antigen for acquired immunity. For example, Minami et al. showed by using PD-L1_11-19_ and PD-L1_41-50_ peptides that PD-L1-specific CTLs were induced in patients with renal cell cancer to kill PD-L1-expressing tumor cells in an HLA-A24-restricted manner [25]. Additionally, Munir et al. demonstrated PD-L1_15-23_ peptide could induce HLA-A2-restricted CTLs specific for PD-L1 [24]. Furthermore, they tried using PD-L1_242-264_ peptide to stimulate HLA-A2-restricted CD8^+^ T-cells and found it impotent. Interestingly, we found helper epitopes in PD-L1_242-264_ peptide, which is an almost the same sequence as the peptide using this study (PD-L1_241-265_). Our defined peptide efficiently induced PD-L1_241-265_-specific HTLs from several healthy donors in an HLA-DR-restricted manner, HLA-DR4 or HLA-DR9, suggesting that PD-L1_241-265_ peptide is promiscuous. We also immunohistologically detected HLA-DR in 8/24 cases (33.3%) of OSCC, indicating that these tumors could be directly targeted by Th1 cells. Therefore, PD-L1-specific HTLs could play a role not only as a helper cell for CTLs but also as a direct killer cell against tumor cells in patients with OSCC, indicating that antitumor immunotherapy targeting PD-L1 would be a promising strategy for OSCC. Moreover, it has been reported that cytotoxicity of HTLs against cancer cells is mediated through the release of effector cytokines such as IFN-γ, perforin, and granzyme B [26]. We indeed found that PD-L1-specific HTLs showed cytotoxicity against tumor cells in vitro with producing granzyme B and inhibited tumor growth in vivo. To our knowledge, this is the first detailed report about PD-L1-specific HTLs.

Since PD-L1 is expressed not only on tumor cells but also on immune cells such as DCs, autoimmune disease caused by PD-L1-specific T-cells must be considered. Shamaila et al. showed PD-L1-specific CTLs had cytotoxicity to autologous DCs expressing PD-L1 [24]. However, in the present study, PD-L1_241-265_-specific HTLs produced less IFN-γ against autologous DCs compared with tumor cells. This discrepancy may be due to the difference in effector functions between HTLs and CTLs. HTLs mainly function in support of CTLs, while CTLs mainly play the role of killer cell. Therefore, the reaction to PD-L1-expressing normal cells including immune cells must be closely monitored in the clinical setting although we did not see high PD-L1 expression in specimens from non-cancerous patients.

PD-L1 is also expressed on myeloid-derived suppressor cells (MDSCs) to regulate auto-immunity [42]. However, in cancer immunotherapy, the existence of MDSCs indicates poor prognosis and treatment efficacy [43] because of their suppressive function against antitumor immune cells. We, therefore, expect that PD-L1-specific HTLs could eliminate MDSCs in a PD-L1-dependent manner, thereby improving the tumor microenvironment. However, we found that PD-L1-specific HTLs had low killing activity against DCs that expressed PD-L1. Therefore, the interaction between PD-L1-specific HTLs and MDSCs should be further investigated.

Although vaccination with TAA has been designed and implemented for many years, its clinical efficacy has not yet been demonstrated despite success in increasing tumor-specific T-cells in treated patients [44, 45]. The remarkable clinical efficacy of immune checkpoint inhibitors demonstrates that the tumor microenvironment is under a more highly suppressive condition than we expected and PD-L1 is one of the key molecules involved in immune suppression. Desired immune responses, therefore, would not be achieved by only vaccinating cancer patients with TAA peptides without blocking immune suppressive signaling, even if immunoadjuvants are simultaneously used. In the current study, we intratumorally injected IFN-β but not IFN-γ in vivo analysis because immunoadjuvants such as poly(I:C) and CpG are often used in clinical settings and mainly induce type I IFNs to enhance immune activity in cancer patients. However, type I IFNs also upregulate PD-L1 molecules in immune cells and tumor cells [46]. This “double-edged sword” effect of type I IFNs is a challenge to be overcome. However, it is a great advantage for a vaccine therapy targeting PD-L1. Indeed, PD-L1-specific HTLs showed higher cytotoxic activity against IFN-γ-pretreated tumor cell lines than untreated tumor cell lines in vitro and intratumoral treatment of IFN-β did not negatively affect the function of PD-L1-specific HTLs for inhibiting tumor growth in vivo. Therefore, although efficacy of the PD-L1 peptide vaccine remains to be evaluated for future, the combination therapy of the PD-L1 peptide with a type I IFN-inducing adjuvant would be a rational strategy for patients with HNSCC.

## Conclusions

In summary, our findings suggest that PD-L1 is a favorable target for patients with HNSCC and the cancer immunotherapy targeting PD-L1 as a tumor antigen would be a rational strategy for HNSCC patients.

## Data Availability

All data generated or analyzed during this study are included in this published article.

## Abbreviations

HNSCC: head and neck squamous cell carcinoma
OSCC: oropharynx squamous cell carcinoma
HPV: papillomavirus
OS: survival
PFS: progression-free survival
PD-1: programmed cell death-1
PD-L1: programmed cell death-1 ligand 1
CTLs: cytotoxic T lymphocytes
HTLs: CD4^+^ helper T lymphocytes
SCC: squamous cell carcinoma
mAb: monoclonal antibody
IFN-γ: interferon gamma
PBS: phosphate-buffered saline
PBMCs: peripheral blood mononuclear cells
DCs: dendritic cells
GM-CSF: macrophage colony-stimulating factor
IL: interleukin
APCs: antigen-presenting cells
TAA: tumor-associated antigen
MDSCs: myeloid-derived suppressor cells
